# Identification of immunotherapy and radioimmunotherapy targets on desmoplastic small round cell tumors

**DOI:** 10.3389/fonc.2023.1104693

**Published:** 2023-04-05

**Authors:** Madelyn Espinosa-Cotton, Hong-Fen Guo, Satish K. Tickoo, Nai-Kong V. Cheung

**Affiliations:** ^1^ Department of Pediatrics, Memorial Sloan Kettering Cancer Center, New York, NY, United States; ^2^ Department of Pathology, Memorial Sloan Kettering Cancer Center, New York, NY, United States

**Keywords:** tumor-associated antigen (TAA), immunotherapy, radioimmunotherapy, bispecific antibodies (BsAbs), T cell engaging bispecific antibodies, desmoplastic small round cell tumor

## Abstract

**Background:**

Development of successful antibody-based immunotherapeutic and radioimmunotherapeutic strategies rely on the identification of cell surface tumor-associated antigens (TAA) with restricted expression on normal tissues. Desmoplastic small round cell tumor (DSRCT) is a rare and generally neglected malignancy that primarily affects adolescent and young adult males. New therapies capable of treating disseminated disease are needed for DSRCT, which is often widespread at diagnosis.

**Methods:**

We used immunohistochemistry (IHC) on fresh frozen surgical specimens and patient-derived xenograft (PDX) tumors and flow cytometry on DSRCT cell lines to evaluate expression of TAAs in these tumors. *In vitro* cytotoxicity assays were used to evaluate the efficacy of T cell-engaging bispecific antibodies (T-BsAbs) directed at these targets. *In vivo*, we used an intraperitoneal xenograft mouse model of DSRCT to test T-BsAbs against several TAAs.

**Results:**

In DSRCT specimens we found widespread expression of B7-H3, EGFR, GD2, HER2, mesothelin, and polysialic acid, clinical targets for which specific antibody therapeutics are available. The expression of B7-H3, EGFR, HER2, and mesothelin was confirmed on the cell surface of DSRCT cell lines. *In vitro* cytotoxicity assays confirmed the efficacy of T cell-engaging bispecific antibodies (T-BsAbs) directed at these targets against DSRCT cells. Remarkably, a HER2xCD3 T-BsAb was capable of completely shrinking established tumors in an intraperitoneal mouse model of DSRCT.

**Conclusions:**

We propose that these TAAs should be further investigated in preclinical models as targets for immunotherapy and radioimmunotherapy with the hope of providing a rationale to extend these therapies to patients with advanced DSRCT.

## Background

Immunotherapy and radioimmunotherapy have the potential to cure advanced cancers. Immune checkpoint inhibitors (ICIs) such as anti-programmed cell death protein 1 (PD-1)/programmed death-ligand 1 (PD-L1) and anti-cytotoxic T-lymphocyte-associated protein 4 (CTLA-4) antibodies have revolutionized treatment for immunologically “hot” tumors including melanoma and have led to long-term survival for a subset of patients. However, immunologically “cold” tumors, including most pediatric tumors, don’t readily respond to ICI monotherapy. Successful immunotherapeutic strategies for these tumors must be capable of inducing an immune response in an environment with few functional tumor-infiltrating immune cells. One notable example of this is the use of the anti-GD2 monoclonal antibodies (mAbs) dinutuximab (Unituxin, United Therapeutics) and naxitamab-gqgk (Danyelza, Y-mAbs Therapeutics) to engage myeloid effectors and natural killer (NK) cells in high-risk neuroblastoma. Alternative strategies include bispecific antibodies (BsAbs) and chimeric antigen receptors (CAR) to redirect polyclonal T cells to kill, as was shown in B cell malignancies and select solid tumor models. Many of these approaches against “cold” tumors have been antibody-based. These immunotherapeutic and radioimmunotherapeutic strategies rely on the identification of cell membrane-bound tumor-associated antigens (TAA).

In order to prevent on-target, off-tumor toxicity, these TAA targets must be restricted in their expression to tumor tissue, with very limited or no expression in normal tissue. We set out to test a panel of NCI high priority TAA candidates for immunotherapy in desmoplastic small round cell tumors (DSRCT), a rare and understudied malignancy that primarily affects adolescent and young adult males (1, 2). Current treatment for DSRCT includes high-dose chemotherapy, whole-abdomen radiation, and surgery, which are ineffective at curing disseminated disease and lead to long-term toxicities. Despite this aggressive approach, the five-year survival rate is 15-30% (3). Developing new strategies to treat disseminated disease is particularly urgent unmet need for DSRCT, since the majority of patients already have metastases at the time of diagnosis, and relapse is common. For these reasons, identifying appropriate TAA in DSRCT that can serve as targets for immunotherapy and radioimmunotherapy is timely (4). Here, we report on tumor expression of TAA in 14 DSRCT surgical specimens using immunohistochemistry (IHC) with a panel of T cell-engaging BsAbs (T-BsAbs) built on the optimized IgG-[L]-scFv platform for clinical development (5, 6) and confirm the anti-tumor efficacy of these T-BsAbs *in vitro* and *in vivo*.

## Methods

### Patient tumors

Tumor specimens were collected with consent from patients at Memorial Sloan Kettering Cancer Center. Specimens were frozen in Tissue-Tek optimal cutting temperature compound (OCT) (Sakura, Torrance, CA, USA) and stored at -80°C. The MSKCC institutional review board (IRB) approved all aspects of the project.

### Immunohistochemistry

OCT-embedded tumors were sectioned at 5 µm thickness using a cryostat and mounted to a glass slide. Slides were stored at -80°C. On the day of the experiment, the immunohistochemical procedure was performed as follows. All steps were performed at room temperature unless otherwise specified. Slides were incubated in a humidified chamber during steps 5-12. For all washing steps, slides were dipped 15x in three sequential baths of fresh PBS.

1. Frozen slides were thawed for 30 minutes, fixed in acetone at -20°C for 30 minutes, then washed.
2. Slides were fixed in 0.1% H_2_O_2_ diluted in PBS for 15 minutes, then washed.
3. Each tissue section was circled using a hydrophobic PAP pen (Abcam, Cambridge, UK).
4. Avidin blocking solution (Vector Laboratories, Burlingame, CA, USA) was dropped onto each tissue section sufficient to cover the section and left for 20 minutes, then washed.
5. Biotin blocking solution (Vector Laboratories, Burlingame, CA, USA) was dropped onto each tissue section sufficient to cover the section and left for 20 minutes, then washed.
6. 10% horse serum diluted in PBS was added to each tissue section and left for 45 minutes, then removed by turning the slide sideways and tapping the side of the slide against a Kimwipe (Kimberly-Clark, Irving, TX, USA).
7. T-BsAbs were diluted to a concentration of 2 µg/mL in 10% horse serum, added to each tissue section, left for 60 minutes, then washed. (For the CD3 staining, a commercial murine IgG1 anti-human CD3 antibody (Stemcell Technologies, Catalog #60011) was used in lieu of a T-BsAb.)
8. Mouse anti-human OKT3 antibody (BioLegend, San Diego, CA, USA) diluted in PBS was added to each tissue section at a concentration of 0.1 µg/mL, left for 30 minutes, then washed. (For the CD3 staining, this step was omitted.)
9. Biotinylated horse anti-mouse IgG antibody (Vector Laboratories, Burlingame, CA, USA) diluted in PBS at 1:500 was added to each tissue section, left for 30 minutes, then washed.
10. VECTASTAIN Elite ABC peroxidase (Vector Laboratories, Burlingame, CA, USA) was added to each tissue section, left for 30 minutes, then washed.
11. DAB substrate (Vector Laboratories, Burlingame, CA, USA) was added to each tissue section, left for two minutes, then washed in running water for five minutes.
12. Slides were dipped 10 times in hematoxylin, then washed in running water for five minutes.
13. Slides were dipped 15 times in 75% ethanol, 15 times in 95% ethanol, then left in 100% ethanol for two minutes.
14. Slides were dipped 15 times in xylene, then left in fresh xylene for two minutes.
15. Three drops of CytoSeal 60 (Richard-Allen Scientific, San Diego, CA, USA) were added to each slide and covered with a glass coverslip.

### Scoring of TAA expression

Stained tissue sections were examined and reviewed by a trained pathologist (Dr. Satish K. Tickoo, MSKCC). Scoring was performed using a modified H-score with a scoring range of 0 to 300 (7). A score of zero was assigned for negative or trace staining, 1 for weak staining, 2 for moderate staining, and 3 for intense staining. Each intensity score was multiplied by the proportion of the tumor cells with that intensity, and added together as such:


Modified H−score=[(0× % score 0)+ (1  × % score 1)+ (2 × % score 2)+ (3 × % score 3)]

### Generation of T-BsAbs

T-BsAbs were generated as previously described (8). Briefly, the IgG-[L]-scFv format used for the T-BsAbs consists of a huOKT3 scFv fused to the c-terminus of the light chain of human IgG1. The T-BsAbs differ in their complementarity-determining regions (CDRs) of IgG1, which confer specificity for each particular TAA. The parental antibody clones used to design CDRs of each T-BsAb are as follows: B7-H3 – 8H9; c-MET – onartuzumab; CD19 – FMC63; EGFR – C225 (cetuximab); GD2 – 3F8; HER2 – 4D5 (traztuzumab); L1CAM – CE7; mesothelin – MORAb-009; polysialic acid – HP35; PSMA – J591.

### Cell culture

JN-DSRCT-1 cells were a gift from the late Dr. William L. Gerald (MSKCC). BER cells were a gift from Dr. Emily Slotkin (MSKCC). SK-DSRCT-2 cells were a gift from Dr. Marc Ladanyi (MSKCC). All cell lines were cultured in RPMI 1640 medium containing L-glutamine (Corning Scientific, Corning, NY, USA) with 10% fetal bovine serum (Gibco, Gaithersburg, MD, USA) and 1% penicillin/streptomycin (Gibco, Gaithersburg, MD, USA). Cells were incubated at 37°C with 5% CO_2_ and passaged two or three times per week.

### Luciferase transduction

A six-well plate was coated with retronectin (20 μg/mL) and incubated overnight at 4°C. Retronectin was aspirated, 3 mL 2% BSA in PBS was added per well, and the plate was incubated for 30° minutes at room temperature. BSA was aspirated and wells were washed once with PBS. 3 mL supernatant from a click beetle red luciferase/tdTomato retrovirus (kindly provided by Vladimir Ponomarev [MSKCC]) was added to each well and the plate was centrifuged at 1240 x g for 90 minutes at 32°C. Viral supernatant was aspirated and 300,000 BER DSRCT cells were added per well. The plate was checked daily and cells were trypsinized and removed two days later when they reached ~80% confluency. Transduction was confirmed by assessing fluorescence of the red fluorescent protein tdTomato by flow cytometry and by incubating the transduced BER cells (BER-luc cells) with D-luciferin (GoldBio, St. Louis, MO, USA) and measuring luminescence using a Synergy H1 Hybrid multi-mode microplate reader (Biotek, Winooksi, VT, USA).

### Flow cytometry

1,000,000 DSRCT cells per well were plated in a 96-well, U-bottom plate and incubated with T-BsAbs (10 µg/mL) in 100 µL cell culture media at 4°C for 60 minutes. Cells were washed twice with PBS, resuspended with PE-conjugated mouse anti-human IgG Fc antibody (BioLegend, San Diego, CA, USA) diluted 1:500 in PBS, and incubated at 4°C for 30 minutes. Cells were washed twice more in PBS, resuspended in 50 µL per well PBS, and flow cytometry was performed using an Attune NxT Flow Cytometer (Thermo Fisher Scientific, Waltham, MA, USA). Data was analyzed using FlowJo software (FlowJo LLC, Ashland, OR, USA).

### Cytotoxicity assays

Standard chromium-release assays were performed to assess cytotoxicity as previously described (9). Briefly, DSRCT cells were incubated with ^51^Cr chromate (Amersham Biosciences, Arlington Heights, IL, USA) for one hour, washed, and plated in 96-well, U-bottom plates. BsAbs and activated human T cells were added and plates were incubated for four hours at 37°C. Plates were centrifuged at 400 x g for five minutes. A gamma-counter (Packard Bioscience Company, Downers Grove, IL, USA) was used to measure radiation in supernatant.

### *In vivo* experiments and animal care

Animal studies were conducted in accordance with Institutional Animal Care and Use Committee guidelines, under protocol 09-05-010. Six to eight-week-old male CIEA BRG C.Cg-*Rag2^tm1Fwa^IL2rg^tm1Sug^*/JicTac mice (Taconic Inc., Hudson, NY, USA) were housed in pathogen-free rooms in MSKCC’s Research Animal Resource Center (RARC) and fed chow containing sulfamethoxazole and trimethoprim (Sulfatrim). Mice were injected intraperitoneally (IP) with 2 x 10^6^ BER-luc or JN-DSRCT-1-luc DSRCT cells in 200 µL PBS in the lower right quadrant of the abdomen near the midline. Initially, 5 mice per experimental group were injected with tumor cells based on previous experiments conducted using T-BsAbs in our lab that suggest this sample size would be sufficiently large enough to determine statistically significant differences in treatment efficacy. Tumors were detectable using an IVIS Spectrum *in vivo* imaging system (PerkinElmer, Waltham, MA, USA) one to two weeks after injection, at which time mice were randomized by tumor size into treatment groups. For tumor imaging, mice were anesthetized using isoflurane, injected retro-orbitally with 100 µL D-luciferin (30 mg/mL) and placed supine in the imager. Not all the mice in the experiment using JN-DSRCT-1-luc cells developed tumors. Mice that had no detectable tumor signal on day 20 were not included in the experiment. Mice were treated once weekly with 20 x 10^6^ activated human T cells injected retro-orbitally, twice weekly with BsAbs injected IP, and once weekly with recombinant human IL-15/IL-15R-alpha injected SC (10). BsAbs were prepared as previously described (11, 12). Tumor growth was monitored once weekly using the IVIS Spectrum *in vivo* imaging system. All mice that were randomized into treatment groups at the beginning of the experiments were included in the data analyses. All cages within an experiment were housed on the same rack in the same room of MSKCC’s RARC. The order of treatments and imaging was varied from week to week to minimize potential confounding. Because one person was responsible for the execution and data analysis of these experiments, blinding was not possible.

### ELISAs for cytokines

DuoSet enzyme-linked immunosorbent assays (ELISAs) were purchased from R&D Systems (Minneapolis, MN, USA). Blood was collected from mice 24 hours after the first administration of activated human T cells and BsAbs, centrifuged at 2000 x g for 10 minutes, and serum was removed and frozen at -80°C for later use. On the day the ELISA assays were performed, serum was thawed at room temperature and diluted 1:10 with reagent diluent. Assays were performed according to manufacturer’s instructions.

### Statistical analysis

Statistical analysis was performed using Graphpad Prism software. One-way ANOVA with Dunnett’s multiple comparison tests were used to compare areas under the curve for tumor growth experiments and cytokine secretion in ELISA experiments. P values < 0.05 were considered statistically significant.

## Results

### DSRCT expresses known clinical targets for immunotherapy and radioimmunotherapy

**Figure 1:**
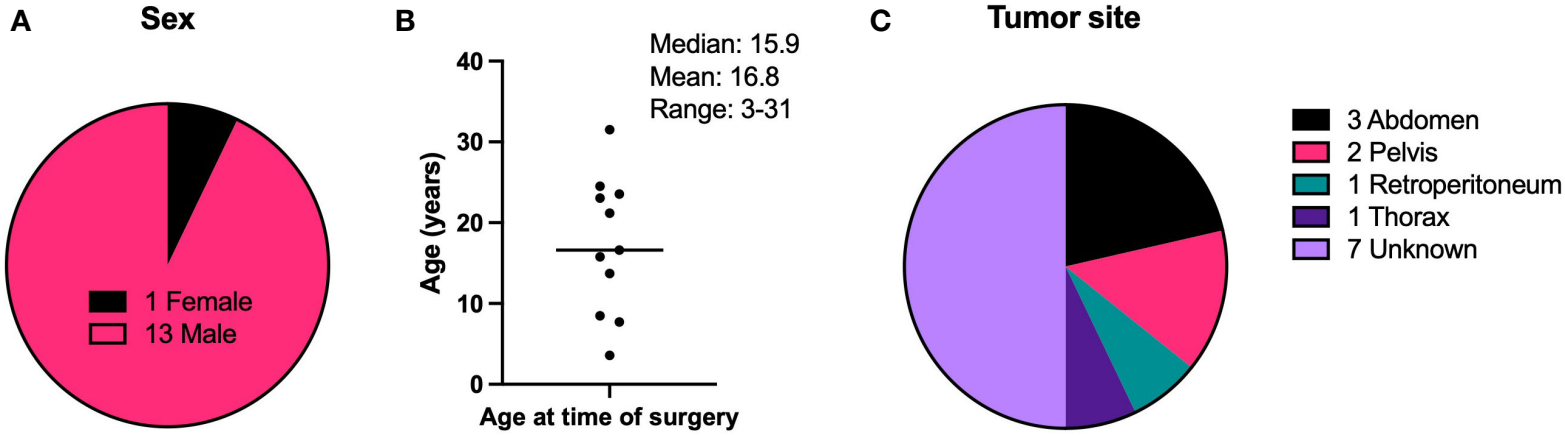
Demographic characteristics of DSRCT patients. **(A)** Biological sex of patients. **(B)** Ages of patients at the time of surgery to remove the tumor specimen used in IHC experiments. **(C)** Anatomical site of the tumor specimen resected during surgery and used in IHC experiments. N = 14.

Tumor specimens were collected from DSRCT patients undergoing surgery at MSKCC. 14 were randomly chosen from a list of 63 archived samples with intact histology. **Figure 1** shows demographic and tumor site information on these patients. 13 of the patients were male and one was female. The median age of all patients at the time of surgery was 16.6 years, the mean was 17.2 years, and the age range of all patients was 3-31 years. For specimens with tumor site information, the majority were found within the abdomen and pelvis, with one each in the retroperitoneum and the thorax. The characteristics of this sample set of tumors is representative of DSRCT tumors in general, which are most commonly found in the abdomen or pelvis of adolescent and young adult males (13, 14).

**Figure 2:**
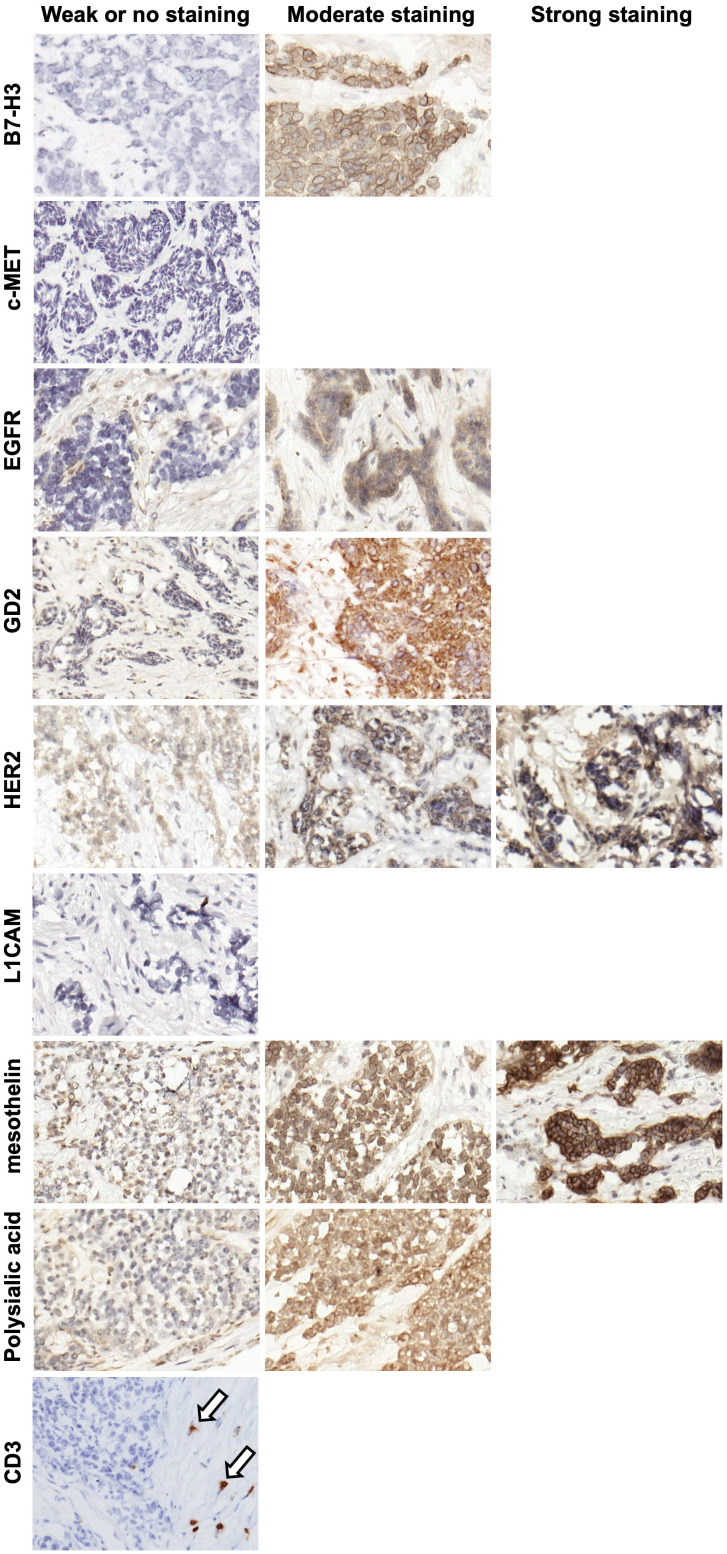
Representative IHC images of DSRCT tumor specimens. Images were taken of DSRCT specimens stained with T-BsAbs (specific BsAb indicated on the left of the images) or commercial anti-human CD3 antibody and scored with a modified H-score system. Specimens deemed to have weak or no staining had H-scores between 0 and 100, specimens deemed to have moderate staining had H-scores between 101 and 200, and specimens deemed to have strong staining had H-scores between 201 and 300. If a representative image is missing from the moderate or strong staining categories, this indicates that no specimens stained in this range for this particular target. Arrows indicate CD3+ cells in the stroma.

**Figure 3:**
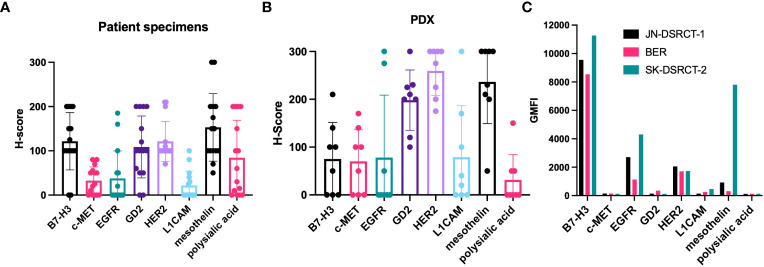
Expression of target antigens in DSRCT specimens, PDX, and cell lines. **(A)** 14 DSRCT specimens were scored for the intensity of staining for each target antigen using a modified H-score system. The scores are plotted individually with dots. The bar shows the mean of the scores of the specimens for that particular target antigen. **(B)** Scores of PDX specimens. **(C)** Three DSRCT cell lines (JN-DSRCT-1, BER, and SK-DSRCT-2) were incubated with T-BsAbs specific for the target antigens of interest. Flow cytometry was performed to measure binding of the BsAbs to the surface of the cells. GMFI = geometric mean fluorescence intensity. Error bars indicate standard error of the mean. N = 14.

IHC was performed to evaluate the expression of known TAA targets for immunotherapy and radioimmunotherapy including B7 homolog 3 (B7-H3) (15, 16), c-Met (17, 18), epidermal growth factor receptor (EGFR) (19, 20), GD2 (21), human epidermal growth factor receptor 2 (HER2) (22), L1 cell adhesion molecule (L1CAM) (23, 24), mesothelin (25, 26), polysialic acid (27), and prostate-specific membrane antigen (PSMA) (28) using target-selective antibodies built using a IgG-[L]-scFv T-BsAb format as previously outlined (5, 9, 11, 12, 29, 30). IHC using commercial antibodies against human CD3 revealed very few CD3-positive cells (T cells) in the tumors which were almost uniformly restricted to the stroma and absent from the nests of tumor cells **(** **Figure 2** **)**. This is consistent with other reports describing DSRCT as an immunologically “cold” tumor (31–33). Importantly, these findings mean that the results of our staining with the IgG-[L]-scFv T-BsAbs are not confounded by the anti-CD3 scFv binding T cells in the tumor, and instead reflect expression of TAAs only. **Figure 2** shows examples of weak (score 0-100), moderate (score 101-200) and strong (score 201-300) staining for each antigen. B7-H3, EGFR, GD2, HER2, mesothelin, and polysialic acid exhibited clear membrane staining with the moderate and strong staining tumors also exhibiting some cytoplasmic staining. In general, there is heterogeneity among tumors for all the TAAs studied. Mean score was lowest for L1CAM followed by c-MET, and EGFR. Four of the antigens examined (B7-H3, GD2, HER2, and mesothelin) had mean scores in the moderate range **(** **Figure 3A** **)**. This staining strategy was repeated on a group of eight DSRCT patient-derived xenograft tumors with similar results **(** **Figure 3B** **)**.

### Expression of immunotherapy and radioimmunotherapy targets on DSRCT cell surface

In order for BsAb-based immunotherapy or radioimmunotherapy to be effective, TAAs must be expressed on the cell surface. To determine whether this was the case for our TAAs, we performed flow cytometry to assess binding of T-BsAbs to DSRCT cell lines. The cells were not permeabilized and therefore BsAb binding could only occur with targets expressed on the outer surface of the cell membrane. **Figure 3C** shows binding of B7-H3, EGFR, GD2, HER2, L1CAM, and mesothelin in at least one of three DSRCT cell lines. B7-H3 showed high cell surface expression in all three cell lines, while expression of EGFR, HER2, and mesothelin, were detected at lower levels in all cell lines tested. GD2 and L1CAM were detectable on the surface of one or two cell lines, respectively, but not all three. There was no expression of c-MET or polysialic acid on any of the three DSRCT cell lines tested. The lack of cell surface c-MET expression was consistent with IHC results in **Figure 2**. Although polysialic acid was found on the surface of DSRCT patient tumor specimens **(** **Figures 2** and **3A** **)**, it were absent from all three cell lines tested. Outgrowth *in vitro* of clones missing polysialic acid with proliferative advantage could explain these differences.

### *In vitro* cytotoxicity of T-BsAbs against DSCRT cell lines

**Figure 4:**
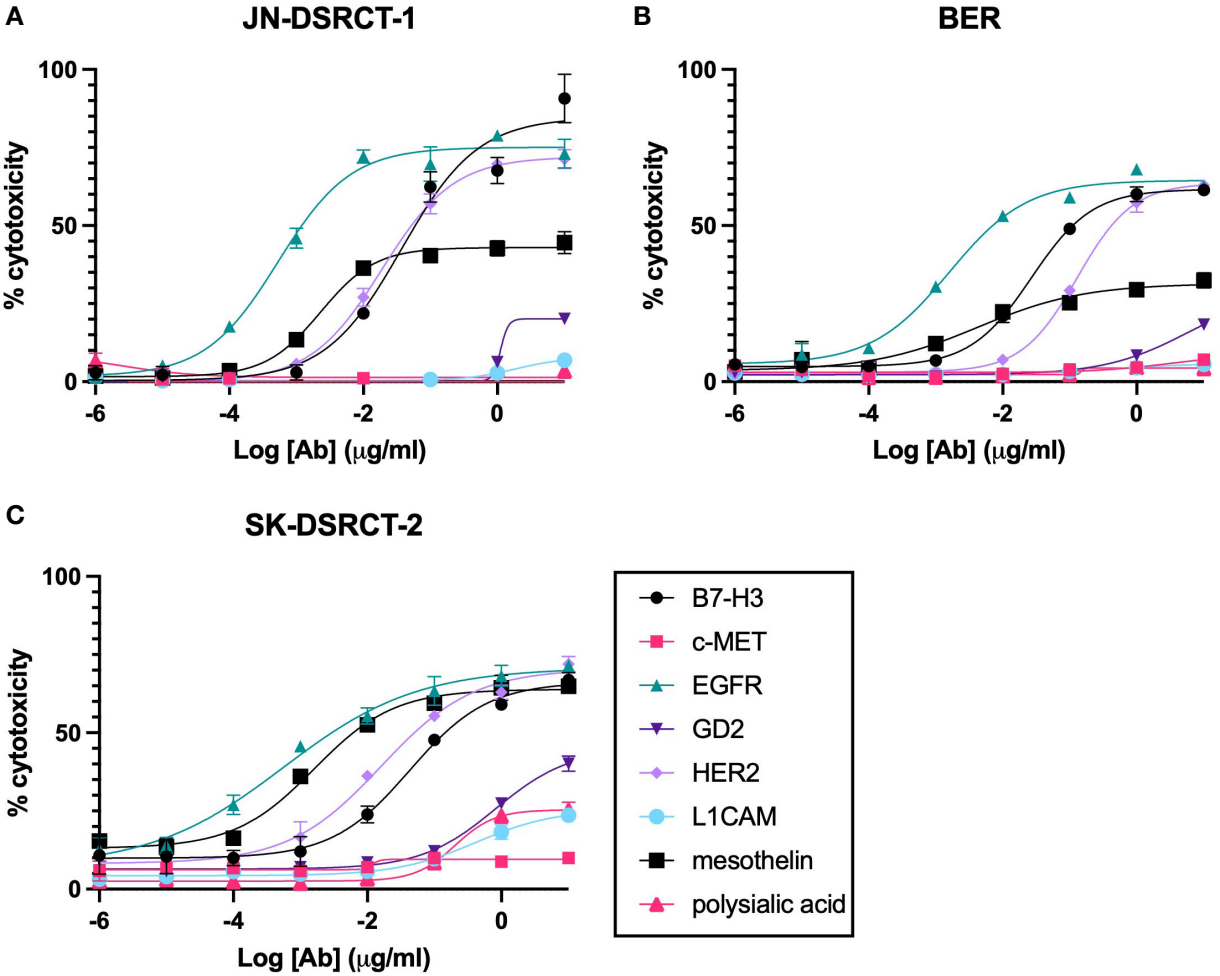
*In vitro* cytotoxicity of BsAbs incubated with DSRCT cells and activated human T cells. Chromium-release assays were performed on cells from three DSRCT cell lines (JN-DSRCT-1 **[A]**, BER **[B]**, and SK-DSRCT-2 **[C]**) incubated with different concentrations of T-BsAbs and activated human T cells. All assays were performed a minimum of three times per cell line. Representative plots are shown. N = three technical replicates per data point.

One way to assess the ability of T-BsAbs to engage cytotoxic T cells and kill tumor cells is by using *in vitro* cytotoxicity assays. We performed standard chromium-release assays using T-BsAbs directed against our TAAs of interest and activated human T cells in culture with three DSRCT cell lines. **Figure 4** shows that the T-BsAbs specific for B7-H3, EGFR, and HER2 were highly cytotoxic in all three cell lines. The mesothelin T-BsAb was moderately cytotoxic in the JN-DSRCT-1 and BER cell lines and highly cytotoxic in the SK-DSRCT-2 cell line. The GD2 T-BsAb showed minimal cytotoxicity at high concentrations, consistent with the low level of GD2 expression on the surface of these cell lines. The c-MET, L1CAM, and polysialic acid T-BsAbs exhibited no or very little cytotoxicity, even at high concentrations. In general, the level of TAA cell surface expression (shown in **Figure 3C**) correlated with the level of *in vitro* cytotoxicity mediated by the corresponding T-BsAbs.

### *In vivo* efficacy of T-BsAbs against intraperitoneal DSRCT xenografts

**Figure 5:**
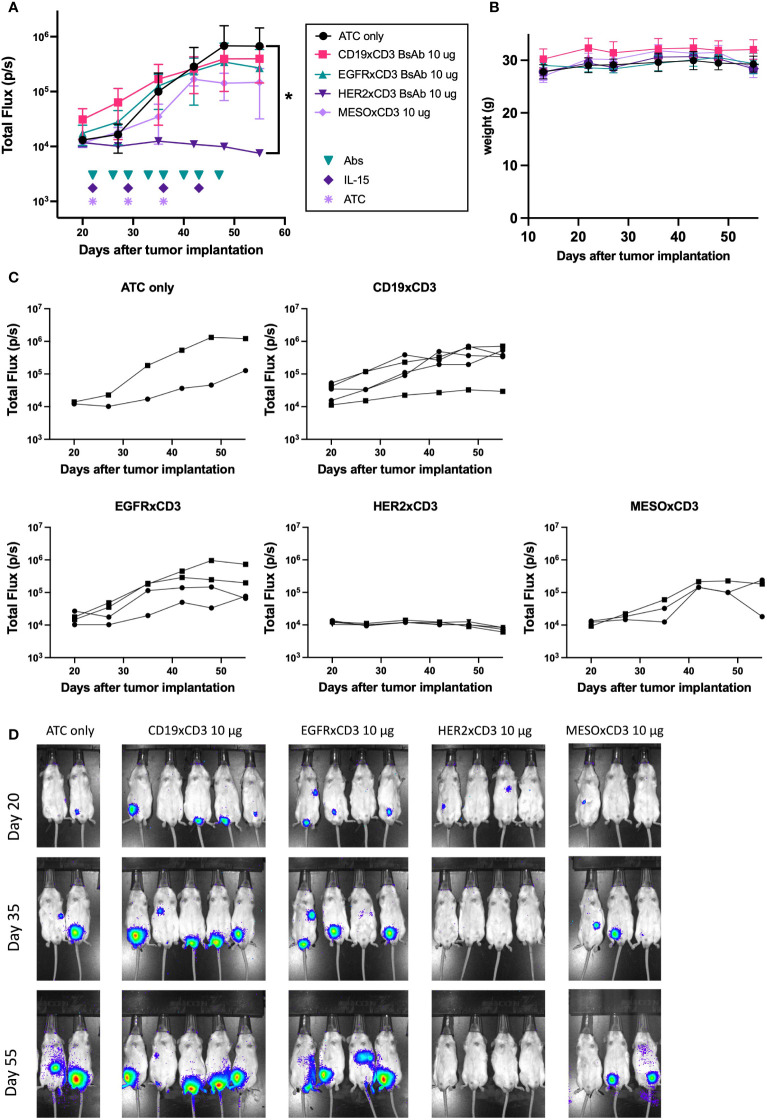
*In vivo* efficacy of T-Bsabs against intraperitoneal JN-DSRCT-1-luc xenografts. 25 mice (five per treatment group) were injected intraperitoneally with 2 x 10^6^ JN-DSRCT-1-luc cells stably transduced with luciferase. Two weeks after injection, tumor establishment was evaluated using an IVIS bioluminescent imager. Mice with visible tumors were treated intravenously with activated human T cells (ATC) once weekly, intraperitoneally with T-BsAbs twice weekly, and subcutaneously with recombinant human IL-15/IL-15R-alpha once weekly. Mice without any visible tumors two weeks after injection were excluded from treatment. A group treated with an irrelevant BsAb (CD19xCD3 BsAb), activated human T cells, and IL-15/IL-15R-alpha was included as a negative control. Tumor growth was monitored weekly using bioluminescent imaging. **(A)** Plot of mean total flux (photons/second), indicating tumor size. **(B)** Plot of weight of mice in each group. **(C)** Plots of each treatment group showing individual mice. Error bars indicate standard error of the mean. **(D)** Mice were injected retro-orbitally with 50 μL D-luciferin (30 mg/mL) one minute before imaging, anesthetized with isoflurane, and situated in a supine position. A 10 second exposure was used for all images and other parameters are identical in all images to allow comparison across groups and timepoints. N = two to five mice per group. * = p < 0.05.

While several of our T-BsAbs were able to engage T cells to destroy tumor cells *in vitro*, the ability to do so *in vivo* should greatly enhance their clinical relevance. To determine *in vivo* anti-tumor efficacy we tested the EGFR (n=4), HER2 (n=4), and mesothelin (n=3) T-BsAbs in an intraperitoneal model of DSRCT (JN-DSRCT-1-luc), which more closely recapitulates human disease than a standard flank xenograft. Mice in these treatment groups were engrafted with human activated T cells (ATC) and given recombinant human IL-15/IL-15R-alpha to support T cell survival *in vivo*. ATC only (n=2) and irrelevant T-BsAb + ATC (CD19xCD3 BsAb, n=5) groups were included as negative controls. **Figures 5A** (showing comparisons between groups of 2-5 mice each) and **5C** (showing tumor growth for all 18 mice individually) show that only the HER2 T-BsAb was able to control tumor growth (p = 0.02 compared to ATC only group). The EGFR T-BsAb performed no better than ATC alone (p = 0.28), and the mesothelin T-BsAb-treated group had one tumor that began to regress around day 40 but ultimately did not statistically differ from the ATC only group (p = 0.09). The HER2 T-BsAb completely ablated tumors by day 27 after tumor implantation, one week after treatment began **(** **Figure 5D** **)**. The lack of *in vivo* anti-tumor efficacy by EGFR and mesothelin T-BsAbs despite their *in vitro* potency was surprising although similar negative results have been seen with other tumor targets, such as B7-H3 (data not shown). This discordance could result from position of the epitope being too distal from the cell membrane, insufficient antigen density or antibody affinity (34, 35). The mice did not lose weight during treatment **(** **Figure 5B** **)** and did not show any of sign of clinical toxicity until around day 60 when signs of graft versus host disease (GVHD - hair loss, skin reddening, weight loss) appeared, at which point the experiment was terminated and the animals were euthanized. This is a known consequence of engrafting activated human T cells into immunocompromised mice.

### *In vivo* efficacy of a HER2xCD3 T-BsAb against aggressive intraperitoneal DSRCT xenografts

**Figure 6:**
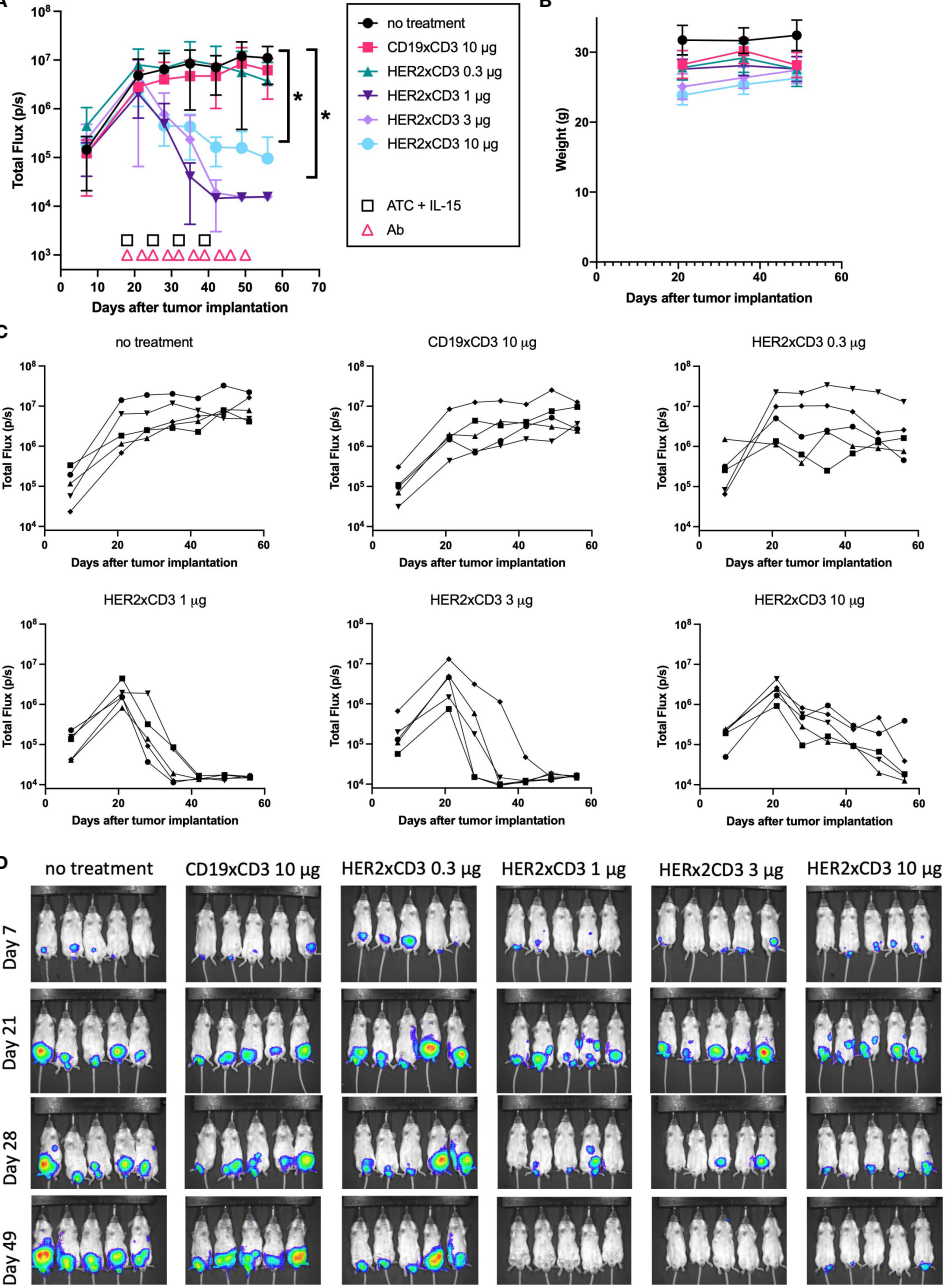
*In vivo* efficacy of a HER2 T-BsAb against intraperitoneal BER-luc xenografts. Mice were injected intraperitoneally with a DSRCT cell line stably transduced with luciferase (BER-luc). Tumor growth was monitored with an IVIS bioluminescent imager. Mice were treated intravenously with activated human T cells once weekly, intraperitoneally with T-BsAbs twice weekly, and subcutaneously with recombinant human IL-15/IL-15R-alpha once weekly. An untreated group (no treatment) and a group treated with an irrelevant BsAb (CD19xCD3), activated human T cells, and IL-15/IL-15R-alpha (CD19xCD3) were included as controls. **(A)** Plot of mean total flux (photons/second), indicating tumor size. **(B)** Plot of weight of mice in each group. **(C)** Plots of each treatment group showing individual mice. Error bars indicate standard error of the mean. **(D)** Mice were injected retro-orbitally with 50 μL D-luciferin (30 mg/mL) one minute before imaging, anesthetized with isoflurane, and situated in a supine position. A 10 second exposure was used for all images and other parameters are identical in all images to allow comparison across groups and timepoints. N = five mice per group. * = p < 0.05.

After observing the striking efficacy of the HER2 T-BsAb against JN-DSRCT-1-luc xenografts, we sought out to evaluate the efficacy of this T-BsAb in a faster-growing model of DSRCT (BER-luc). In this experiment, average total flux (photons/second) was around 10^6^ cells for BER-luc tumors at the beginning of treatment, versus 10^4^ cells with JN-DSRCT-1-luc in the previous experiment **(** **Figures 5A** and **6A** **)**. A total of 30 mice were used in this experiment. Four dose levels of HER2 T-BsAb were tested (0.3, 1, 3, and 10 μg per dose, n=5 mice per treatment level) and all tumors treated with at least 1 μg/dose began to shrink after the second injection of ATC and third dose of BsAb **(** **Figures 6A** **)**. **Figure 6C** shows the tumor growth of each individual mouse. No treatment and irrelevant T-BsAb + ATC groups (CD19xCD3 BsAb) were included as negative controls (n=5 mice each). The lowest dose of HER2 T-BsAb had no effect on tumor growth at any point during the experiment compared to the negative control untreated group (p = 0.99). The two intermediate dose levels (1 μg and 3 μg/mouse) completely ablated tumors by day 49 after tumor implantation (p = 0.03 and 0.07, respectively, vs no treatment control), with no luciferase signal detectable by day 49 **(** **Figure 6D** **)**. The highest dose level (10 μg/mouse) also shrunk established tumors (p = 0.04 vs no treatment control), however, 3/5 mice in this group still had detectable luciferase signal in their abdomens on day 49. None of the mice exhibited signs of toxicity and no significant weight loss was observed **(** **Figure 6B** **)**. Around day 50 the mice treated with ATC began to exhibit signs of GVHD at which point the experiment was terminated and the animals were euthanized.

### Effects of T-BsAbs on cytokine secretion from human T cells *in vivo*

**Figure 7:**
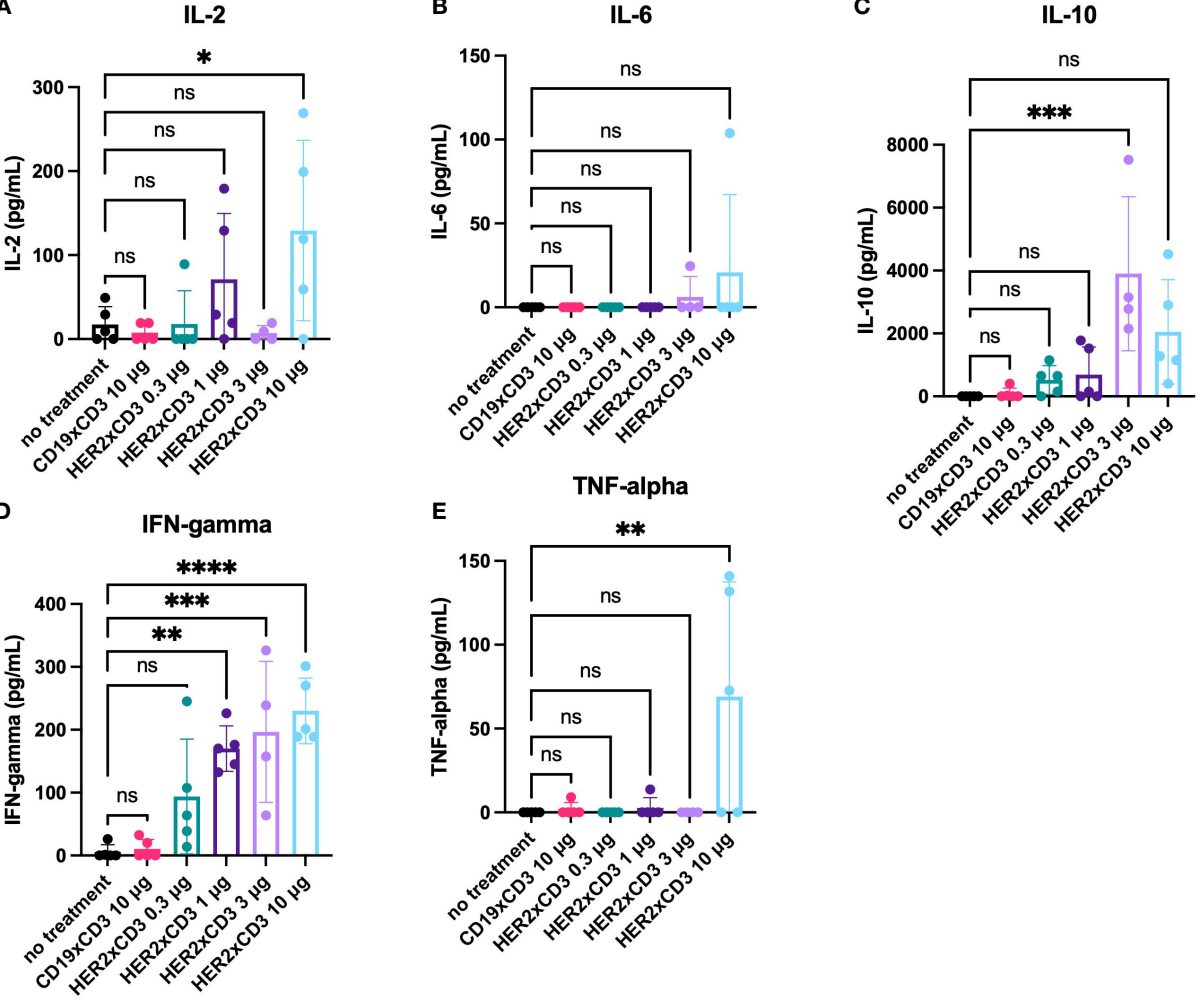
Serum cytokine levels in mice treated with HER2xCD3 BsAb. 24 hours after the first injection of activated human T cells and T-BsAbs, blood was collected from mice retro-orbitally. Serum was separated and assayed for the presence of human cytokines (**[A]** IL-2, **[B]** IL-6, **[C]** IL-10, **[D]** IFN-gamma, **[E]** TNF-alpha) using ELISA kits. Values from individual mice are plotted using dots and the mean of each group is plotted using a bar. Error bars indicate standard error of the mean. N = five mice per group. * = p < 0.05, ** = p < 0.01, *** = p < 0.001, **** = p < 0.0001 ns, not significant.

In an effort to evaluate the effects of different doses of HER2 T-BsAb on the activity of T cells *in vivo*, blood was taken from the mice 24 hours after the first administration of T-BsAbs and activated human T cells and ELISAs were performed on the serum. Interferon gamma (IFN-gamma) was detected in the blood of all mice treated with HER2 T-BsAb in a dose-dependent trend **(** **Figure 7D** **)**. The overall levels of interleukin 2 (IL-2) were low and near the limit of detection for the assay, though IL-2 in the 10 μg HER2 T-BsAb group was significantly higher compared to the untreated group (p = 0.026) **(** **Figure 7A** **)**. Only two mice, one in each of the highest dose groups, had detectable interleukin-6 (IL 6) in their serum at this timepoint **(** **Figure 7B** **)**. Interleukin 10 (IL-10) was detectable in many of the mice treated with HER2 T-BsAb but was highest in the two highest dose groups **(** **Figure 7C** **)**, with the 3 μg group being significantly higher compared to the no treatment control (p = 0.0003) and the 10 μg group approaching significance (p = 0.05). IFN-gamma was significantly elevated in the 1 μg, 3 μg, and 10 μg groups (p = 0.0016, 0.0006, and <0.0001, respectively). Finally, tumor necrosis factor alpha (TNFα) was significantly upregulated in the 10 μg HER2 T-BsAb group compared to the untreated control (p = 0.0039) **(** **Figure 7E** **)**. It was detected at high levels in 3/5 mice in the 10 μg HER2 T-BsAb group but only detectable at very low levels in two other mice in different groups **(** **Figure 7E** **)**. TNFα has been described as the “master of T cell exhaustion” and has been shown to influence T cells towards an exhausted phenotype (36). This finding may explain why 10 μg HER2 was less effective than 1 μg or 3μg. This phenomenon has been noted by other researchers studying biologic drugs and is the basis for the rational move away from determining the maximum tolerated dose (MTD) in early stage clinical trials and towards instead identifying the optimum biologic dose, which may well be lower than the MTD (37).

## Discussion

The results of our IHC staining in a panel of 14 DSRCT surgical specimens supports the findings previously reported by our lab and others (16, 38–40) that B7-H3 and GD2 are expressed in DSRCT in both the cell membrane and stroma (16, 38, 39). Using a semi-quantitative scoring system, B7-H3 and GD2 were both abundant in DSRCT surgical specimens and PDXs. Furthermore, our work confirms previous reports in the literature of EGFR, HER2, and mesothelin expression in smaller sample sets of DSRCT specimens assayed by IHC (41–43).

We have previously described BsAbs for targeting T cells and radioisotope using this panel of TAAs in other solid tumors (5, 8, 11, 12, 30, 44–46). Before applying these BsAbs to DSRCT, IHC was used to confirm their membrane expression. Once extracellular expression of TAAs is confirmed, both immunotherapy and radioimmunotherapy can be exploited. One important finding among the panel of DSRCT was the near absence of CD3+ T cells (**Figure 2**), which is typical for immunologically “cold” tumors, such as most solid pediatric tumors. The absence of CD3 positivity allows us to use T-BsAb in IHC without the concern of nonspecific background staining. Once TAA expression is confirmed in human specimens, these targets can now be exploited for treatment with IgG, T-BsAb, DOTA-BsAb, or CAR constructs. One DSRCT patient included in a basket trial for HER2-positive sarcomas had stable disease for 14 months following treatment with HER2 CAR T cells (47). As molecular profiling of tumors becomes more commonplace clinicians should be able to direct patients to basket trials using these types of immunotherapeutic strategies. This could especially benefit DSRCT patients, whose tumors are so rare that few clinical trials exist for them specifically. One clinical trial that did enroll DSRCT patients specifically evaluated the safety of intraperitoneal ^131^I-8H9, a radioiodinated anti-B7-H3 antibody (48). This therapy was shown to be safe and well-tolerated and has since progressed to Phase II. Another type of radioimmunotherapy, a three-step pre-targeted radioimmunotherapy (PRIT) system, has been successfully applied using the same IgG-[L]-scFv platform as our T-BsAbs (45). More recently, the self-assembling disassembling (SADA) 2-step PRIT system was successfully employed to deliver ablative doses of beta-emitters or alpha-emitters without any evidence of myelosuppression, nephrotoxicity, neurotoxicity, or hepatotoxicity (49). These PRIT strategies have increased therapeutic indices to levels not previously possible, allowing high radiation doses to be delivered to the tumor with minimal off-tumor effects compared to traditional radio-conjugated antibodies.

Another recent development in immunotherapy and radioimmunotherapy is the use of compartmental (e.g. intraperitoneal) delivery. By delivering the agent intraperitoneally it is possible to achieve high tissue levels restricted to body compartments while sparing systemic exposure, decreasing the on-target, off-tumor toxicities often encountered with IgG radioimmunoconjugates. This strategy was employed in the ^131^I-8H9 clinical trials. DSRCT is particularly well-suited for compartmental therapy due to its propensity to spread within the peritoneal cavity. Here, we demonstrated the efficacy of delivering T-BsAbs intraperitoneally against DSRCT IP xenograft tumors. While the HER2xCD3 T-BsAb was effective at ablating established DSRCT xenografts, this strategy may prove less effective in human patients, whose tumors tend to be much more heterogenous than cell line-derived xenografts. For T-BsAbs, employing a dual-target approach may overcome tumor heterogeneity while also increasing the potency against tumor cells and reducing bystander side effects. For radioimmunotherapy, using beta-emitters (which have longer [mm] path lengths than alpha emitters) may be employed to overcome heterogeneity.

## Conclusions

In summary, we have identified several TAAs that are highly expressed in DSRCT. T-BsAbs directed against these TAAs were effective at directing T cell to kill tumor cells *in vitro*, and one T-BsAb (HER2xCD3) was remarkably effective at ablating established and disseminated IP xenografts *in vivo*. We propose that these TAAs should be further investigated in preclinical models as targets for immunotherapy and radioimmunotherapy with the hope of providing a rationale to extend these therapies to patients with advanced DSRCT.

## Data availability statement

The original contributions presented in the study are included in the article/Supplementary Material. Further inquiries can be directed to the corresponding authors.

## Ethics statement

The studies involving human participants were reviewed and approved by Memorial Sloan Kettering Cancer Center Institutional Review Board. Written informed consent to participate in this study was provided by the participants or the participants' legal guardian/next of kin. The animal study was reviewed and approved by Memorial Sloan Kettering Cancer Center Institutional Animal Care and Use Committee.

## Author contributions

ME-C designed and performed the experiments, interpreted the results, and wrote the manuscript. H-FG generated the T-BsAbs used in the experiments. ST reviewed the IHC staining and assisted with interpretation of results. N-KC assisted in interpreting experimental results and writing the manuscript. All authors read and approved the final manuscript.
